# Ultra-wide field and new wide field composite retinal image registration with AI-enabled pipeline and 3D distortion correction algorithm

**DOI:** 10.1038/s41433-023-02868-3

**Published:** 2023-12-19

**Authors:** Fritz Gerald P. Kalaw, Melina Cavichini, Junkang Zhang, Bo Wen, Andrew C. Lin, Anna Heinke, Truong Nguyen, Cheolhong An, Dirk-Uwe G. Bartsch, Lingyun Cheng, William R. Freeman

**Affiliations:** 1 Jacobs Retina Center, University of California, San Diego, CA USA; 2 Viterbi Family Department of Ophthalmology and Shiley Eye Institute, University of California, San Diego, CA USA; 3 Division of Ophthalmology Informatics and Data Science, Viterbi Family Department of Ophthalmology and Shiley Eye Institute, University of California, San Diego, CA USA; 4 Department of Electrical and Computer Engineering, University of California, San Diego, CA USA

**Keywords:** Anatomy, Education, Medical imaging

## Abstract

**Purpose:**

This study aimed to compare a new Artificial Intelligence (AI) method to conventional mathematical warping in accurately overlaying peripheral retinal vessels from two different imaging devices: confocal scanning laser ophthalmoscope (cSLO) wide-field images and SLO ultra-wide field images.

**Methods:**

Images were captured using the Heidelberg Spectralis 55-degree field-of-view and Optos ultra-wide field. The conventional mathematical warping was performed using Random Sample Consensus—Sample and Consensus sets (RANSAC-SC). This was compared to an AI alignment algorithm based on a one-way forward registration procedure consisting of full Convolutional Neural Networks (CNNs) with Outlier Rejection (OR CNN), as well as an iterative 3D camera pose optimization process (OR CNN + Distortion Correction [DC]). Images were provided in a checkerboard pattern, and peripheral vessels were graded in four quadrants based on alignment to the adjacent box.

**Results:**

A total of 660 boxes were analysed from 55 eyes. Dice scores were compared between the three methods (RANSAC-SC/OR CNN/OR CNN + DC): 0.3341/0.4665/4784 for fold 1-2 and 0.3315/0.4494/4596 for fold 2-1 in composite images. The images composed using the OR CNN + DC have a median rating of 4 (out of 5) versus 2 using RANSAC-SC. The odds of getting a higher grading level are 4.8 times higher using our OR CNN + DC than RANSAC-SC (*p* < 0.0001).

**Conclusion:**

Peripheral retinal vessel alignment performed better using our AI algorithm than RANSAC-SC. This may help improve co-localizing retinal anatomy and pathology with our algorithm.

## Introduction

In retinal practice, aside from fundus examination, diagnoses have been reliant on the use of digital imaging as most retinal imaging modalities are non-invasive and have fast acquisition, not only in 2D but also 3D [1]. The clinical fundus examination is currently used in conjunction with other wide-field imaging in patients with retinal diseases such as diabetes, sickle cell, tumours, peripheral choroidal neovascularization, and many more. The common modalities used are Fluorescein Angiography (FA), Indocyanine Green Angiography (ICGA), Fundus Autofluorescence (FAF), and Optical Coherence Tomography (OCT). Multimodal imaging has been increasingly popular in ophthalmology, particularly in the retina. The images that these devices provide are critical in the decision-making of clinicians for the diagnosis and management of certain ocular diseases. A study by the DRCR Retina Network group noted an association between predominantly peripheral lesions and the risk of worsening diabetic retinopathy using ultra-wide field imaging [2]. The Heidelberg Spectralis (Heidelberg Engineering, Heidelberg, Germany) uses a confocal scanning laser ophthalmoscope (cSLO) and captures images at three wavelengths: short wavelength (486 nm), which captures the surface of the retina, medium wavelength (518 nm) which captures the retinal vascular and inner retinal layers, and long wavelength (815 nm) which captures the RPE and other deep layers [3]. The Optos devices (Optos plc, Dunfermline, United Kingdom), on the other hand, use a scanning laser ophthalmoscope (SLO) and capture images at two wavelengths: red laser (635 nm) and green laser (532 nm) [4]. These two imaging modalities are commonly used in the retinal clinic to provide insight into the patient’s retina status and document certain pathologies. A study by Muftuoglu et al. [5]. concluded that the multicolour (MC) imaging from Heidelberg Spectralis provided superior epiretinal membrane detection compared to conventional fundus photo imaging, which was primarily attributed to the green wavelength of the MC imaging. Commonly, retinal imaging can be performed using a scanning laser ophthalmoscope (SLO) at a wider angle, but most of these machines do not have a built-in OCT. The standard of care in OCT is typically confocal SLO (cSLO) imaging. However, different instruments have different wavelengths, hence different information. Certain instruments are better steered so that if there are peripheral lesions to be seen with an OCT, it can be done with a few manoeuvres. The standard clinical practice is to compare a lesion seen in two imaging modalities by bringing them up on a computer monitor and doing a side-by-side manual comparison. For example, suppose a choroidal tumour was imaged using a 55-degree montage of one MC imaging modality and another one from a colour photograph. In that case, these two composite images will be compared using a side-by-side method. This is time-consuming and may have inaccuracies in co-localizing regions of interest. An automated method to do this would be clinically beneficial and more rapid and accurate than manual methods.

Artificial intelligence (AI) is a general term implying the use of a computer to model intelligent behaviour with minimal human intervention. It is considered a branch of engineering in this generation. Its application in medicine has two branches: physical and virtual. The physical component is represented by robots to aid the physicians or surgeons and patients. The virtual component is represented by Machine or Deep Learning. Deep learning is represented by mathematical algorithms that improve learning through experience [6]. Deep learning has been introduced in other medical specialties like cardiology, dermatology, and neurology [7]. In ophthalmology, deep learning has been applied for medical imaging analysis in detecting various ocular conditions using mainly fundus photographs and optical coherence tomograph [8]. However, there are some limitations of deep learning-based models. If the models fail, unnecessary additional interventions or misdiagnosis may lead to poor outcomes [9]. That is why training the AI is essential to improve the analysis.

Cavichini et al. [10]. compared the conventional mathematical warping (Modality Independent Neighborhood Descriptor [MIND]) and a new AI method in aligning two types of retinal images taken in two different imaging modalities, i.e., colour fundus photographs and infrared scanning laser ophthalmoscope with narrow angles of view primarily of the posterior pole of the eye. They discovered their AI method superior to conventional warping at the posterior pole. This led us to use wider imaging modalities of different specifications to better understand the retinal periphery, as there has been no published literature regarding overlay of UWF images.

The purpose of our study was to compare a new AI method to conventional warping (Random Sample Consensus, Sample and Consensus set [RANSAC-SC]) in the alignment of retinal vessels at the retinal periphery using machines with different optical pathways, resolutions, and wavelengths: ultra-wide field (Optos) and wide-field (Heidelberg Engineering) images. We selected two imaging systems with different optics and wavelengths by performing an AI montage in the Heidelberg images to get the full view of the retina and overlay it with the ultra-wide field (Optos) image. Our group observed the quality of overlay in RANSAC-SC vs. our AI algorithms (Outlier Rejection Convolutional Neural Network [OR CNN] and OR CNN + Distortion Correction [OR CNN + DC]).

## Methods

### Study participants

This study was approved by the Institutional Review Board of the University of California San Diego in California, USA. Data collection and analysis were conducted according to the Principles of the Declaration of Helsinki and complied with the Health Insurance Portability and Accountability Act of 1996. The patient’s consent was obtained as per institution protocol, and all the data that were collected were anonymized.

Participants included in this study were consecutive patients that were seen at the Jacobs Retina Centre, University of California San Diego, from July 2021 until September 2021. Participants had retinal imaging done using Heidelberg HRA + OCT Spectralis System version 1.11.2.0 (Heidelberg Engineering, Heidelberg, Germany) and Optos Optomap Monaco P200TE software version 3.1.0.12 (Optos plc, Dunfermline, United Kingdom).

### Image acquisition and processing

We collected the Ultra-wide field images (UWF), the wide field (WF) images, and generated the WF composite images in the following way: The UWF images were captured by the Optos ultra-wide field retinal imaging device with the colour Optomap image modality and the standard image view (200° internal angle = ~135° conventional nomenclature, single capture). For each eye, we took one UWF image. The WF images were captured using the Heidelberg Spectralis device (55° field-of-view [FOV]) with the following settings: Multicolour mode, ART = 15, High-speed mode and 3 × 3 matrix activated to capture nine fields of the retina. A total of nine WF images were captured in one eye.

A total of nine (9) 55-degree FOV images using the Heidelberg system and one single Optos image were captured in one eye. The Heidelberg steered images, when montaged, comprised a field of view ranging from 110–120 degrees. In this paper, we used the term ultra-wide field (UWF) for Optos images and wide-field (WF) for Heidelberg images.

To generate the WF composite image, we set the UWF image as a fixed reference image and then align each WF image independently to the UWF image. Finally, we fused all WF images from the same eye into one composite image. We applied Gaussian blur to the masks of the WF images to obtain smooth transitions at the edges of the WF overlaying areas.

### Alignment algorithm

Our proposed pipeline for UWF-WF/UWF-Composite WF retina image alignment is an AI-based method and follows a feature-based registration procedure, including feature detection, description, matching as well as outlier rejection. The outlier rejection step is crucial in later transforming the retina image for accurate alignment. Therefore, we compared our AI implementation with the classic RANSAC-SC [11] method in this work. Compared with our previous work in Zhang et al. 2021 [12], in order to align WF images taken from different angles with a UWF image on different regions, we propose a distortion correction algorithm with 5 degrees of freedom for the WF camera to better eliminate the distortion at the outer region of the UWF images. Furthermore, in order to generate and accurately align the composite WF image, we propose a new warping strategy that first warp UWF towards WF to determine the best relative pose between two cameras and then compute the inverse transformation to warp all of the WF images towards the UWF modality.

**Fig. 1:**
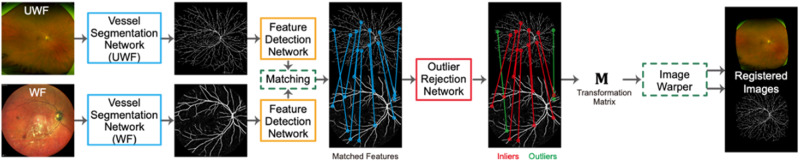
Proposed one-way forward registration procedure. The proposed one-way forward registration procedure including CNNs for vessel segmentation, feature detection, description, and outlier rejection, respectively. CNN Convolutional Neural Network.

In our proposed method (Fig. 1), we first extract vessel maps from both input UWF and WF images by two vessel segmentation convolutional neural network (CNN), respectively. The vessel segmentation CNN for the UWF modality is based on a model pre-trained on a public UWF dataset by Ding et al. [13]. Then, we set up a joint segmentation and deformable registration training procedure similar to the one published by Zhang et al. [14]. to train the vessel segmentation CNN for the WF modality. The segmentation knowledge stored in the pre-trained CNN for UWF can be migrated into the WF segmentation CNN by a deformable alignment network.

After vessel maps are extracted from both input images, we adopted a SuperPoint network [15] as the feature detection and description CNN to extract features from them. The SuperPoint Network has been pre-trained on a synthetic grayscale image dataset and will output key-point coordinates and their respective descriptions for each vessel map. Then, the detected features of both images are matched with each other based on bi-directional consensus, i.e., a feature in UWF should be the best match of a feature in the WF, and vice versa for the same pair of features.

Afterward, we used an outlier rejection CNN [16] to estimate inliers and their weights from the matched features. It takes as input the coordinates of all matched features and outputs weights for each. It has been pre-trained on a retinal registration dataset for Colour Fundus and Infrared images [17], and then fine-tuned on a small UWF-to-WF alignment dataset [12]. We specifically compared AI-based method with the previous RANSAC-SC method, which is specially designed to align two UWF images.

Finally, a transformation matrix was estimated from the matched features and weights by weighted least square (WLS) estimation. We applied second-order polynomial transformation to warp the WF images to align with the UWF image according to the matched inlier key points:

$${[{u}_{i},{v}_{i}]}^{T}={M}_{{poly}}{[{m}_{i},{n}_{i},{m}_{i}^{2},{n}_{i}^{2},{m}_{i}{n}_{i},1]}^{T}$$

where $\left[{u}_{i},{v}_{i}\right]$ and $\left[{m}_{i},{n}_{i}\right]$ are keypoint locations in UWF and WF images, respectively. Here, ${M}_{{poly}}$ is the 2$\times$6 transformation matrix.

**Fig. 2:**
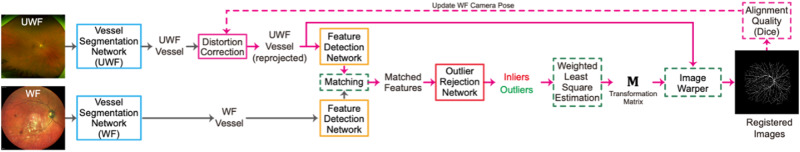
The proposed iterative 3D camera pose optimization process. We first reproject the UWF using the distortion correction function based on the current estimate of WF camera pose, then estimate the transformation model based on the WF and the reprojected UWF images to warp the reprojected UWF image again. Next, compute the alignment quality (Dice) and finally update the WF camera pose for the next iteration. UWF ultra-wide field, WF wide-field.

Due to the perspective distortion in the peripheral area of the UWF image, the above 2D registration method was unable to model the distortion nor perfectly align the two images, even given perfect feature matchings. Therefore, after the CNNs have been set up in the forward 2D registration procedure, we proposed a distortion correction algorithm based on iterative 3D camera pose optimization to incorporate 3D eyeball shape information into the alignment process to improve alignment quality, as shown in Fig. 2. After the distortion correction algorithm, the two images can share similar levels of distortions which can be more easily modelled by 2D-to-2D transformation matrices. As shown in Fig. S1, we followed the assumptions of stereographic projection and spherical eyeball for UWF images, projected the 2D WF image grid onto a 3D sphere, and re-projected it onto the 2D imaging plane of the UWF image based on the WF camera pose. The camera pose is determined by 5 extrinsic parameters of the camera:

$${x}_{{cam}}:\left(x,y,z\right){{{{{\rm{;}}}}}}\, {R}_{{cam}}:({\theta }_{x},{\theta }_{y})$$

where $x,{y},{z}$ are positions in 3D in the coordinate shown in supplemental Fig. S1, ${\theta }_{x},{\theta }_{y}$ are rotations of the camera in x and y directions. To be more specific, the grid $\left(m,n\right)$ of the WF image is first converted to the 3D world coordinate $({m}^{{\prime} },{n}^{{\prime} },{f{{\hbox{'}}}})$ and projected onto the 3D retina surface $({m}^{{\prime \prime }},{n}^{{\prime \prime }},z)$ according to the camera pose. Then the 3D coordinates on eyeball are projected to the UWF image plane and converted to UWF pixel coordinate $({m}_{p},{n}_{p})$. The UWF image can be warped to the same distortion level with the WF image according to the difference between the original and the projected grid:

$${I}_{{uwf}-{warped}}={STN}\left({I}_{{uwf}},\left({m}_{p},{n}_{p}\right)-\left(m,p\right)\right)$$

The 3D eyeball is assumed to be a sphere with radius one, and the UWF camera is placed at the centre of cornea, i.e., (0, 0, −1) in the world coordinate. The UWF image plane is $z=1$ and the WF image plane’s centre is also assumed to be on $z=1$ while its orientation is determined by the WF camera pose. The distortion correction algorithm and the CNNs (2D pipeline) were concatenated into an iterative optimization process (Fig. S2), where we search for an optimized WF camera pose based on the alignment performance between the WF image and the warped UWF image.

Then, we used the optimized camera pose and the same 3D projection model to inversely warp the WF image and project the key point locations on it to the UWF image plane. Finally, the 2D alignment matrix was calculated from the UWF key points and the projected WF key points and warped the WF image to UWF again in 2D. Single warped WF images were then assembled to the composite image to be aligned with UWF image.

### Composite image generation algorithm

For each eye, there is one UWF image with 8–9 WF images. After the WF images and the corresponding binary mask were warped to the UWF image through the 3D projection and 2D alignment, we summed all the WF images from one eye along with the masks. The masks’ boundary was smoothed by Gaussian blurring to get a smoother transition between WF images in the final composite images. Finally, the summed image was divided by the summed mask to generate the composite image. Figure S3 shows the registration of nine 55-degree FOV WF images (A and B) with corresponding UWF image (C).

### Implementation details

The UWF images have pixel resolution of 4000 × 4000. The resolution of the original WF images is 768 × 768 before montaging and varies after montaging. The images were later expanded to the same size as the UWF images (interpolated) for wrapping and composite image generation. Our dataset (446 pairs) was randomly separated into set 1 (237 pairs) and set 2 (209 pairs). For fold 1-2, we tested our algorithm on set 1 and train on set 2. For fold 2-1, we tested set 2 and train on set 1.

The segmentation network was pretrained on colour fundus images [17] and fine-tuned on our dataset. We used learning rate 1e-3 and trained for 2000 epochs. The key point detection network (Superpoint) is pretrained on a hybrid dataset with colour fundus and infrared reflectance retina images in the same way as previously done [17]. For the outlier rejection network, we used learning rate 1e-4 and trained for 1000 epochs. We used Adam optimizer for both the segmentation and outlier rejection network training.

### Grading method

We used a modified version of Dice score in this paper, which is defined as

$${Dice}\left({I}_{1},{I}_{2}\right)=\frac{2\cdot \sum {{pixelwise}}_{\min }\left({I}_{1},{I}_{2}\right)}{\sum {I}_{1}+\sum {I}_{2}}$$

where ${I}_{1}$ and ${I}_{2}$ are two vessel maps in range [0,1].

**Fig. 3:**
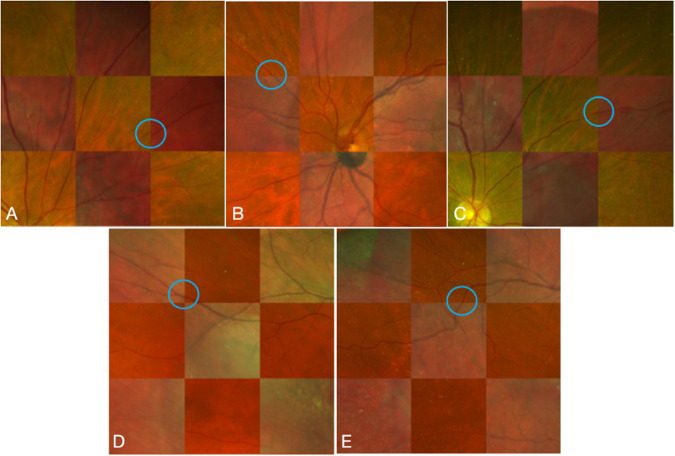
Sample grading evaluation of eyes analysed. Grade 1 (**A**) = > 1 vessel width difference, Grade 2 (**B**) = > ^1^⁄_3_ vessel width difference, Grade 3 (**C**) = ^1^⁄_3_ - ^1^⁄_2_ vessel width difference, Grade 4 (**D**) = ^1^⁄_3_ or less vessel width difference, Grade 5 (**E**) = perfect alignment.

Each overlaid image was presented as a checkerboard composed of alternating Heidelberg multicolour and Optos pseudocolor images. To avoid bias in terms of decision-making from the Likert 5 scale using the generated algorithms RANSAC, OR CNN, or OR CNN + DC, the alignment of the peripheral vessels was graded independently by a masked grader (AL) from the overlaid images. For each overlaid image, an unmasked reviewer (FK) assigned a specific box from the checkerboard with a visible vessel in each adjacent box closest to the optic nerve, which was located to the periphery outside the vascular arcades. The unmasked reviewer located these points at the superotemporal, superonasal, inferotemporal, and inferonasal periphery. When there were multiple vessels seen per box, the unmasked grader assigned a specific vessel to grade. The alignment of vessels was based on the grading from the previous literature by Cavichini, et al. [10]. Grade 1 = > 1 vessel width difference, Grade 2 = > ^1^⁄_2_ vessel width difference, Grade 3 = ^1^⁄_3_ − ^1^⁄_2_ vessel width difference, Grade 4 = ^1^⁄_3_ or less vessel width difference, Grade 5 = perfect alignment (Fig. 3). The masked graders and unmasked reviewer viewed the images in one high-quality widescreen monitor for uniformity. The results from the masked graders were tabulated and analysed by the unmasked graders (FK, LC).

### Statistical analysis

Each composite image interest was graded by the same retina specialist three times using three different composing algorithms. The goodness of retinal vessel alignment at the peripheral retinal vessel arcade was graded into Likert 5 scales. Scales of vessel alignment goodness were compared among three composing algorithms by modelling the probabilities of having a higher scale value within an ordinary generalized estimating equation (GEE). All statistical analysis was performed using SAS software version 9.4.

## Results

We computed the Dice scores between the UWF and the warped WF images based on their respective vessel maps. When using the independent WF images (no composite), the Dice scores are 0.3407/0.4950/0.5133 for fold 1-2 and 0.3343/0.4654/0.4831 for fold 2-1 (RANSAC-SC/OR CNN/OR CNN + DC). When considering the WF composite images, the Dice scores are 0.3341/0.4665/0.4784 for fold 1-2 and 0.3315/0.4494/0.4596 for fold 2-1 (RANSAC-SC/OR CNN/OR CNN + DC). (Table 1) Our distortion correction algorithm requires all the previous steps to be deterministic so that we can search for the best camera extrinsic parameters under a deterministic 3D relationship. However, the result generated by RANSAC is random, bringing major noise to the algorithm. Therefore, we claim that our distortion correction algorithm is not suitable for the RANSAC method.

**Table 1:** Experiment results from different methods.

*Dice*	Single Image		Composite Image	
Method	Set1	Set2	Set1	Set2
RANSAC-SC [11]	0.3407	0.3343	0.3341	0.3315
OR CNN [14]	0.4950	0.4654	0.4665	0.4494
OR CNN + DC	0.5133	0.4831	0.4784	0.4596

*RANSAC-SC* Random Sample Consensus, Sample and Consensus sets, *OR CNN* Outlier Rejection Convolutional Neural Network, *OR CNN* *+* *DC* Outlier Rejection Convolutional Neural Network + Distortion Correction.

**Fig. 4:**
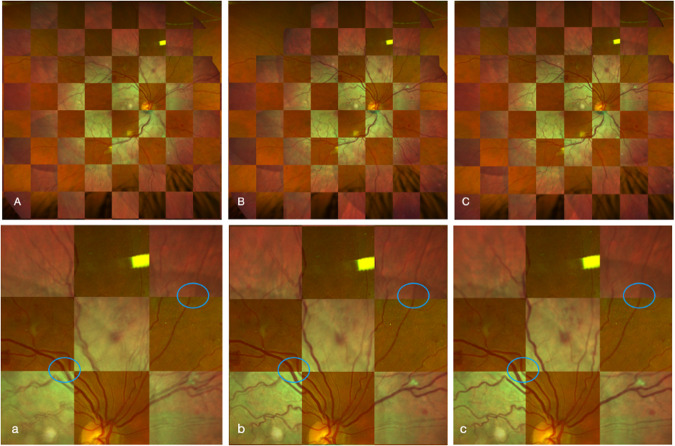
Sample Heidelberg and Optos checkerboard overlay image. Sample Heidelberg and Optos checkerboard overlay image of RANSAC-SC (**A**), OR CNN (**B**), and OR CNN + DC (**C**) with its corresponding magnified image of alignment (a, b, and c, respectively). Images using the AI algorithms OR CNN and OR CNN + DC showed more aligned images than RANSAC-SC, as seen from the encircled vessels. RANSAC-SC Random Sample Consensus, Sample and Consensus sets, OR CNN Outlier Rejection Convolutional Neural Network, OR CNN + DC Outlier Rejection Convolutional Neural Network + Distortion Correction.

Two hundred and twenty boxes from 55 eyes were analysed per algorithm. This yielded a total of 660 boxes. GEE modelling demonstrated that the image composed using our AI algorithm OR CNN or OR CNN + DC has significantly better retinal vessel alignment than the images composed by the RANSAC-SC (Odds ratio 3.6, *p* < 0.0001; Odds ratio 4.8, *p* < 0.0001, respectively). For comparison of AI algorithm OR CNN with OR CNN + DC, OR CNN + DC is superior (Odds ratio 1.3, *p* = 0.0013). A sample comparison between our AI algorithms and RANSAC-SC is shown in Fig. 4. Regarding retinal vessel alignment using the Likert 5 grading scales, the images composed using our algorithm OR CNN + DC have a median rating of 4 (out of 5). In contrast, images composed using RANSAC-SC have a median rating of 2. This means that the alignment of retinal vessels was highest using our algorithm (5 being perfect alignment).

## Discussion

Artificial intelligence in the retina has been increasingly popular worldwide. In 2018, Stevenson et al. [18]. used AI to implement a photo-screening pathway for different retinal diseases. Other groups [19, 20] used deep learning systems, which accurately distinguished retinal pathologies in real time. Aligning the vascular landmarks between two imaging modalities will be important to accurately co-localize different retinal pathologies, particularly in the periphery of the retina.

Our group used these two imaging modalities since wide-angle imaging is also becoming more popular as peripheral retinal or choroidal lesions may also be noted that may warrant further evaluation. Visualization of retinal or choroidal lesions may be different using different imaging modalities. A study from our group by Muftuoglu et al. [21]. compared the characteristics of retinal and choroidal lesions using colour fundus photography and multicolour imaging. We found that the infrared light of the MC imaging underestimates the extent of choroidal lesions by 33%. An innovation of the present study shows that using AI, we can accurately align and overlay images from different types of wide-angle imaging cameras to evaluate lesions outside the posterior pole of the retina. This will be useful when different instruments with different specifications are used.

The ability to colocalize lesions will be an important part of the management. For both natural and medical images, there are always mismatched features that will affect the registration performance. Both the AI model and RANSAC are trying to determine those mismatched features (i.e., outliers) and estimate the transformation matrix using the reliable features (i.e., inliers), yet the AI model has better capability in finding outliers because it can be trained and adapted to the data distribution of retinal images. While RANSAC is a fully randomized method independent of data, its performance is worse. Although the Optos camera captures an approximately 135-degree ultra-wide field image (135° external is the conventional nomenclature and is equal to 200° internal angle measured from the centre of the eye) [22], the ellipsoid mirror provides a different aspect ratio in the periphery than in the centre. Hence, this promotes minimal distortion along the periphery [23]. Our goal was to align the retinal peripheral images captured from the Heidelberg Spectralis to the Optos images using the retinal vasculature as a landmark using our algorithm and compare it with the conventional mathematical warping.

RANSAC is one of the most well-known algorithms because of its robustness and simplicity in solving geometric estimation problems in datasets containing outliers [24, 25]. Our algorithm provided a better alignment in the retinal vasculature than the conventional warping. When comparing the different algorithms, dice coefficients showed better scores using our algorithm; there was mild improvement when OR CNN was used and a vast improvement when DC was added. When retinal vessel alignment scoring was performed, there was a much greater improvement in retinal vessel alignment noted. The clinical scoring we used focused on the peripheral retina, which showed better improvement when OR CNN + DC was used. The checkerboard presented in our study provided certain number of points per human checker, i.e., certain points per retinal image to check, and our results are highly statistically significant.

Theoretically, the centre area (macula) will always have better alignment than the peripheral area because the centre area does not have (perceivable) distortion between the two modalities to correct. Our study evaluated the peripheral retinal vessel alignment using our AI algorithm (OR CNN + DC), which showed superiority over the conventional method (RANSAC-SC). This could help identify lesion sizes (e.g., melanoma, nevus) and monitor progression to understand the pathology better using two imaging modalities. Our method should aid in a better comparison of lesions in the anterior retina. This can be the first step toward further analysis of retinal peripheral imaging, as other imaging modalities may provide insight into retinal architecture. A better understanding of other pathologies by overlaying an optical coherence tomography (OCT) to an algorithm like ours may properly co-localize lesions or abnormalities to the extent that close monitoring or treatment may be warranted. The distortion correction method proposed previously [12] was designed for the case where MC images are in the centre of the fovea. In that case, we assume the relative camera pose (UWF to MC) is mainly different in the distance to the eye while they roughly remain on the same axis. Therefore, we only use one parameter to model the difference between the image-capturing model for both modalities. Regarding the distortion correction model of the present study, since more MC images are in the peripheral retina, we assumed that there are more variations in the relative camera pose. Therefore, we used five parameters to describe the camera pose difference, which can cover more perspective distortions. Ideally, there are six parameters to describe the relative camera pose for perspective projection. We left one dimension, i.e., rotation, to be solved by the following 2D image warping process. Traditional feature descriptors and the work of DeTone et al. [15]. were designed on natural images of similar appearance. However, the variations in modalities will reduce the reliability of their results. Therefore, we transformed multi-modal retinal images into the same modality, i.e., vessel maps, for the following feature detection, increasing the reliability of feature detection results.

To the best of our knowledge, this is the first study to perform an overlay of multicolour wide-field imaging to a pseudocolor ultra-wide field imaging as these two imaging modalities are more commonly used as they are convenient and fast, which lessens the patient burden, especially for uncooperative or unstable patients.

In conclusion, our AI algorithm improved vessel alignment in the retinal periphery compared to RANSAC-SC. This may aid in the future analysis of peripheral changes or pathologies and will allow precise alignment and overlay of peripheral retinal images using a variety of different devices. Other current imaging modalities – adaptive optics, optical coherence tomography/angiography, fluorescein angiography, microperimetry – may benefit from co-localizing retinal anatomy and pathologies.

## Summary

### What was known before

- Wide-angle imaging, as well as high-resolution posterior pole imaging, are increasingly important in understanding and treating retinal disease
- It is difficult to precisely register and compare such images taken on different retinal imaging platforms

### What this study adds

- This study shows that with the use of artificial intelligence, such images can be precisely overlayed and compared to better understand pathology across the entire retina

### Supplementary information

Figure S1

Figure S2

Figure S3

## Data Availability

All data in the current study are available from the corresponding author (WRF) upon reasonable request.
