# Supplementary figures and images for: Ultra-wide field and new wide field composite retinal image registration with AI-enabled pipeline and 3D distortion correction algorithm
Source: Eye (Lond). 2023 Dec 19;38(6):1189–95. doi: 10.1038/s41433-023-02868-3 (PMC11009222; doi:10.1038/s41433-023-02868-3)

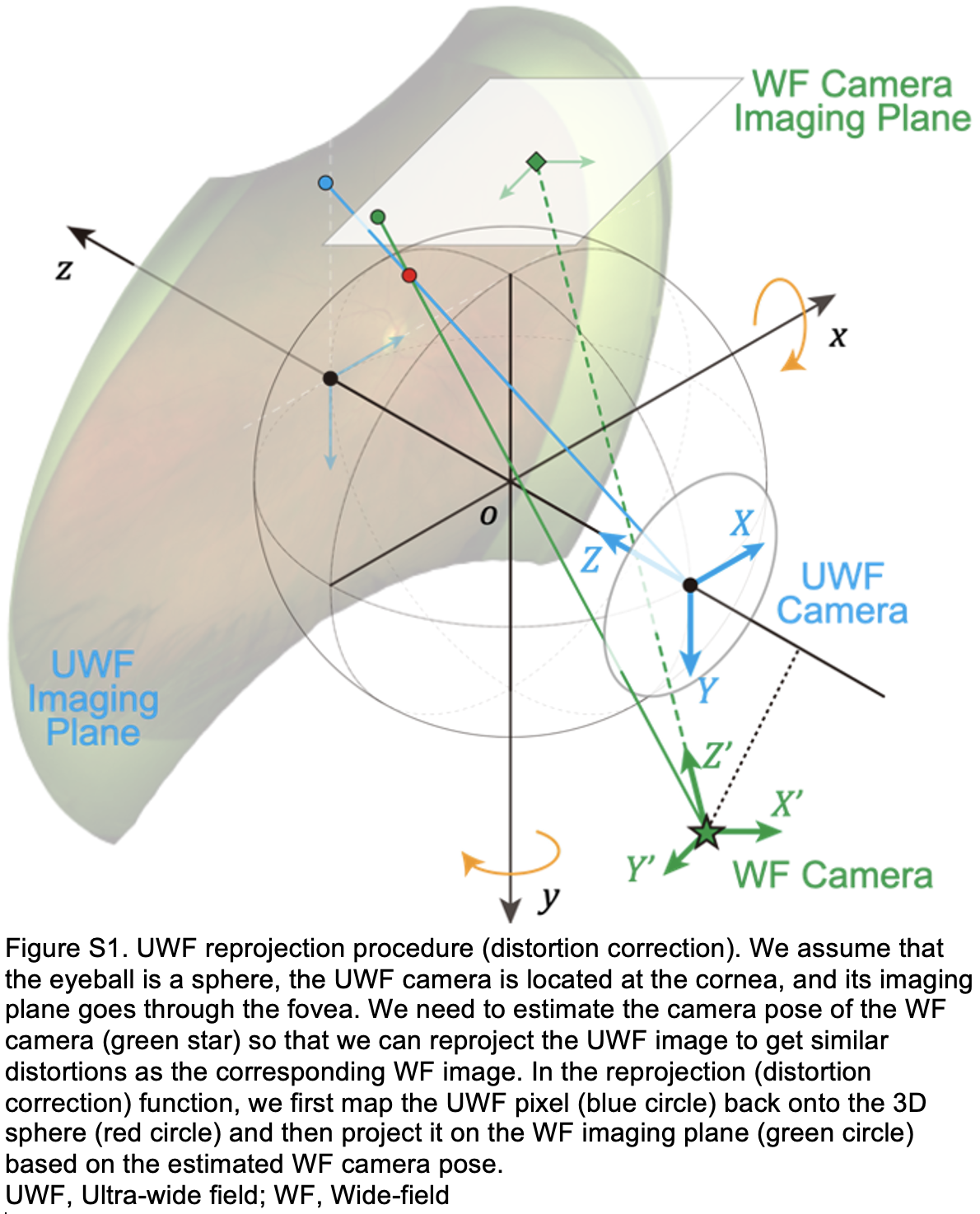

Supplement: Supplementary file 1 — Figure S1 [file 41433_2023_2868_MOESM1_ESM.tif]

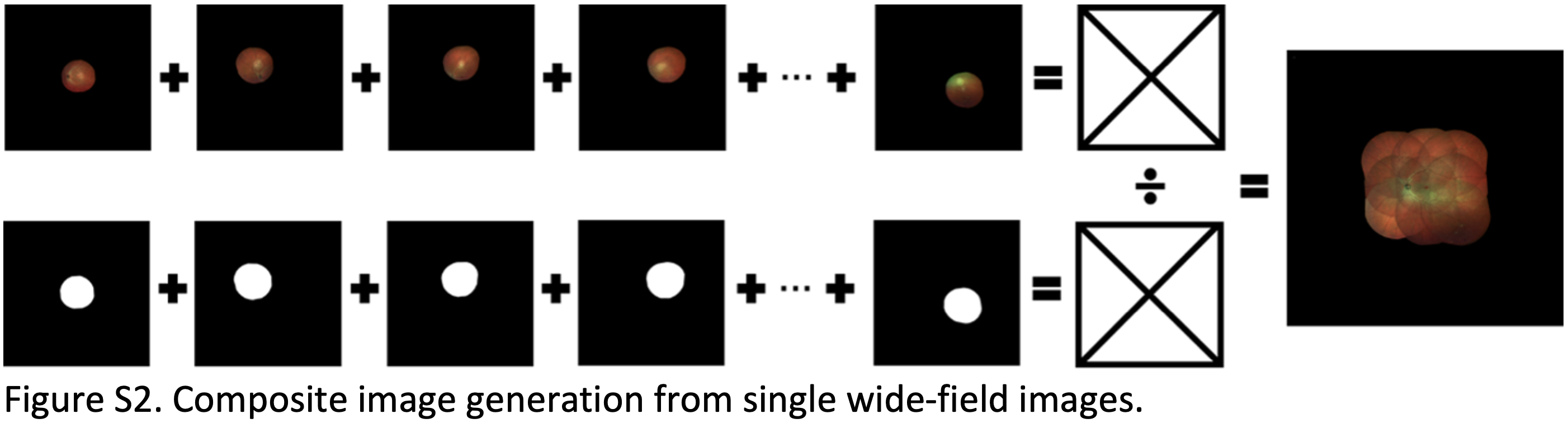

Supplement: Supplementary file 2 — Figure S2 [file 41433_2023_2868_MOESM2_ESM.tif]

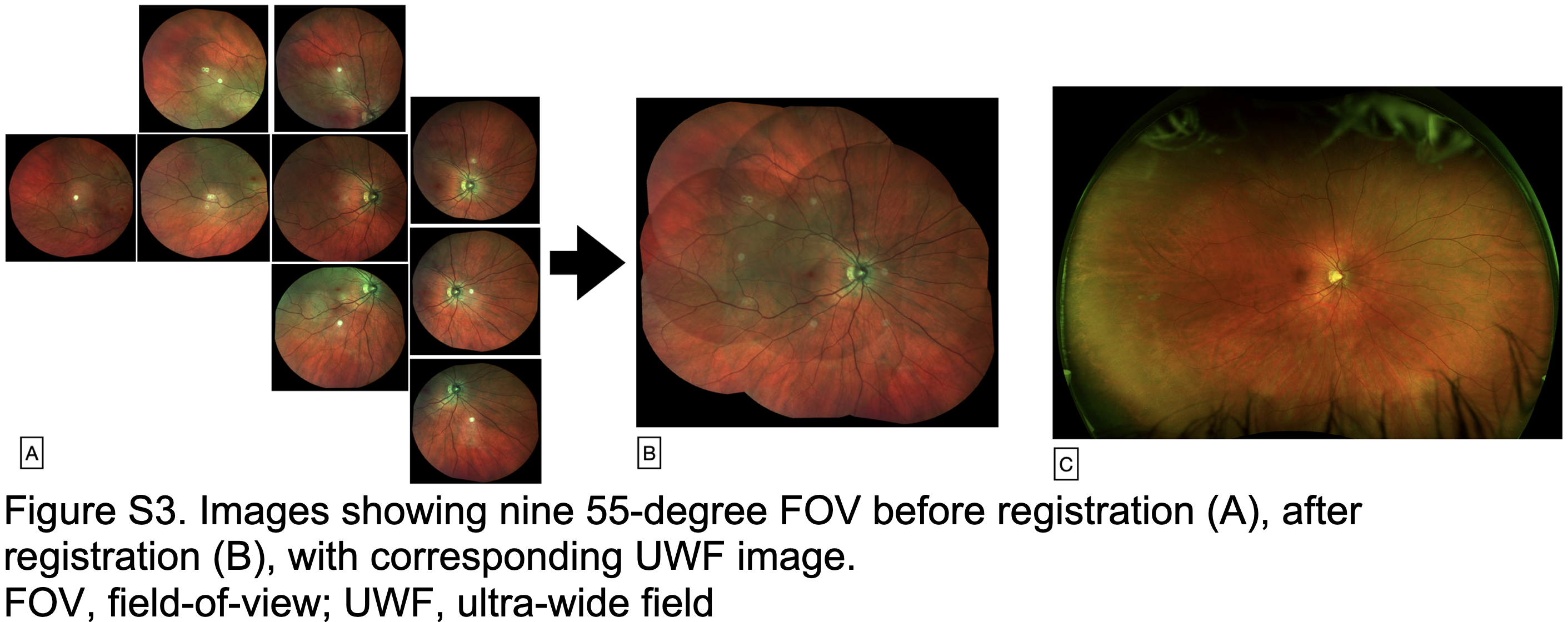

Supplement: Supplementary file 3 — Figure S3 [file 41433_2023_2868_MOESM3_ESM.tif]
